# Toward an evolutionary ecology of (in)equality

**DOI:** 10.1098/rstb.2022.0287

**Published:** 2023-08-14

**Authors:** Eric Alden Smith, Jennifer E. Smith, Brian F. Codding

**Affiliations:** ^1^ Department of Anthropology, University of Washington, Seattle, WA 98195, USA; ^2^ Department of Biology, University of Wisconsin Eau Claire, 105 Garfield Avenue, Eau Claire, WI 54702, USA; ^3^ Department of Anthropology and Archaeological Center, University of Utah, Salt Lake City, UT 84112, USA

**Keywords:** egalitarian, hierarchy, humans, inheritance, mammals, wealth

## Abstract

Inequality is increasingly recognized as a major problem in contemporary society. The causes and consequences of inequality in wealth and power have long been central concerns in the social sciences, whereas comparable research in biology has focused on dominance and reproductive skew. This theme issue builds on these existing research traditions, exploring ways they might enrich each other, with evolutionary ecology as a possibly unifying framework. Contributors investigate ways in which inequality is resisted or avoided and developed or imposed in societies of past and contemporary humans, as well as a variety of social mammals. Particular attention is paid to systematic, socially driven inequality in wealth (defined broadly) and the effects this has on differential power, health, survival and reproduction. Analyses include field studies, simulations, archaeological and ethnographic case studies, and analytical models. The results reveal similarities and divergences between human and non-human patterns in wealth, power and social dynamics. We draw on these insights to present a unifying conceptual framework for analysing the evolutionary ecology of (in)equality, with the hope of both understanding the past and improving our collective future.

This article is part of the theme issue ‘Evolutionary ecology of inequality’.

## 1. Introduction

Our goal in assembling this issue is to explore the insights that evolutionary ecology can bring to the study of inequality, while encouraging transdisciplinary dialogue and a pluralistic view of relevant ideas. The forms and dynamics of inequality have long been central concerns in several social sciences, including anthropology and archaeology, economics, history, political science and sociology. In biology, the study of dominance and reproductive skew are well-established fields of inquiry [1–3]. This theme issue draws on these existing research traditions, exploring ways they might enrich each other, or perhaps be synthesized. The papers herein investigate mechanisms shaping variation in inequality, paying attention to ways in which inequality is resisted or avoided as well as developed or imposed. Most of them do so within the framework of evolutionary ecology and examine the utility of social science concepts such as wealth, property, social power and institutions.

Defining inequality is not straightforward, as its meaning depends on context, ranging from colloquial use to economic analysis to mathematics. In empirical research, inequality is typically defined through quantitative measures such as Gini coefficients or skew indices, with the factors shaping these variables left open to investigation. While straightforward, this lumps variation in a given trait (e.g. accumulated wealth or reproductive success) due to genetic endowment and random accidents with that due to social interactions. Accordingly, for present purposes we define inequality as those differences that are imposed on individuals (or classes of individuals) by structural features of a social system. Thus, inequality as used here focuses on that subset of phenotypic variation shaped by social structures—reinforced within or across generations—that privileges some individuals over others.

Furthermore, our concern is with systematic, socially driven inequality in wealth and the effects this has on differential power (social influence or control over conspecifics), well-being (health, stress, mortality, etc.), reproduction and ultimately fitness. We define wealth as attributes or possessions that contribute to well-being or fitness. Forms of wealth can be material (resources, such as food or territory), relational (social networks) or embodied (knowledge, skill) [4–7]. Note that in this view, power or social influence is viewed as a *consequence* of underlying wealth inequalities, although more power can also contribute to subsequent wealth accumulation.

## 2. Comparative inequality: theory and evidence

For this theme issue, we formulated several key questions about inequality (as defined above):

(a) What factors shape variation in inequality within and across species?
(b) How and why is inequality in human societies similar and different from other mammals, including our primate relatives?
(c) Why was persistent institutionalized inequality in *Homo sapiens* rare for most of our species' existence, yet spread widely in recent millennia?
(d) What are the consequences of inequality for differences in social influence, nutritional state, well-being, survival and reproduction?

**Figure 1.**
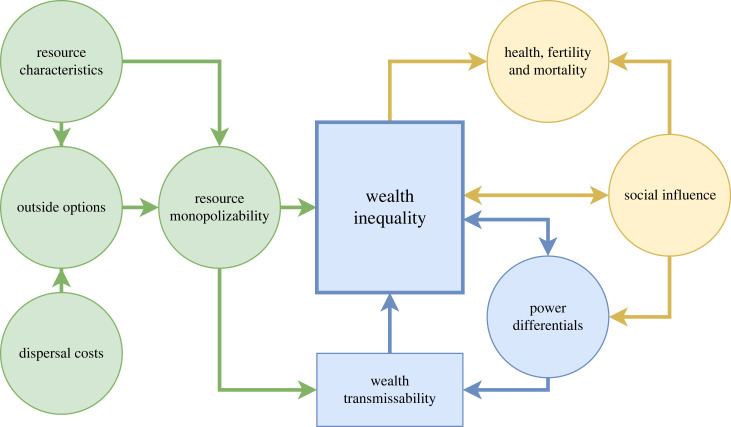
Some causes and consequences of wealth inequality. The left side of the diagram includes ecological and economic drivers of inequality, while the right side lists major biological outcomes. Arrows indicate the primary pathways delineated in theoretical and empirical research, although additional possible pathways and feedback loops are omitted in the interest of legibility. (Online version in colour.)

The following discussion of the papers in this issue and related research is organized around these questions. We build on this information to synthesize a conceptual framework for understanding the evolutionary ecology of (in)equality (figure 1).

### (a) Factors shaping variation in inequality

Considerable research across multiple disciplines has contributed insights into the variables and mechanisms influencing variation in inequality. Within the evolutionary ecology paradigm, perhaps the most frequently invoked drivers are ecological parameters such as resource density, predictability, and patchiness or clumping that facilitates control by a subset of individuals within a society [8,9]. In this issue, theoretical and cross-cultural analyses highlight the effect of these variables in shaping the form and degree of inequality [10–12]. Their importance is given further support in archaeological case studies, which also implicate Malthusian population dynamics involving competition for diminishing resources [13,14].

From a strategic or game-theoretical standpoint, another key variable is the available alternatives to being subordinated—defined as wielding relatively low power within a social group [15,16]. These alternatives, often termed ‘outside options’, might involve joining a different group, migrating to an ‘empty’ locale, or even actively resisting forms of oppression (e.g. [17]). Which options are feasible, and their associated consequences for individuals, is determined to a considerable degree by local or regional socioecological conditions. Theory supported by empirical evidence indicates that switching groups or migrating to greener pastures has lower odds of succeeding as population density increases and/or as resource-quality gradients steepen, as discussed in several papers in this issue [11,18,19]. Resistance to subordination can be costly for both subordinates and dominants [20], and the balance of these costs will shape the outcome; in some cases, threats may be enough to exact a better deal (reduced inequality in resource sharing) for subordinates, as examined in concession models of reproductive skew [21,22] or bargaining models in social science [23,24].

Factors facilitating or impeding wealth inheritance can play a prominent role in shaping inequality for both humans [4,25,26] and non-humans [5,7]. Although any of the three forms of wealth noted above can be transmitted to descendants, material forms are generally more successfully inherited. These can include arable land, livestock, durable goods, resource patches, burrows, food caches, nesting sites and the like, as discussed in several papers in this issue [12–14,20,27,28]. However, embodied wealth such as skills or knowledge passed down from parents [27] or parental investment in offspring condition [29,30] can be important as well, contributing to developmental origins of inequality [30]. This differential access early in life can impose lifetime consequences for individuals [31]. Likewise, relational wealth such as social support from kin or allies can play critical roles in some cases [7,21,28,32].

Finally, factors that do not fit readily into the tripartite wealth typology appear to shape variation in inequality in particular cases. Specifically, the ways in which hierarchy can facilitate decision-making and other forms of collective action have received prominent attention in the animal behaviour literature on movement decisions [33], as well as analyses of variation in political forms of human societies [24,34].

### (b) Comparing humans and other species

One goal of this theme issue is to help strengthen theoretical and empirical linkages in research on inequality across biological and social science disciplines. We are well aware of the difficulties and potential pitfalls in comparing human and non-human behaviour, particularly when it concerns complex patterns of behaviour such as property/territory inheritance [35] and systems of domination and subordination [36]. Nevertheless, there is much to be gained from careful and nuanced sharing of concepts between evolutionary biology and social sciences. The benefits of such cross-fertilization are exemplified by the adaptation of game theory to evolutionary contexts by biologists, and in turn the near replacement of classical game theory with evolutionary game theory in economics. Such mutual influence is central to several papers in this issue [11,18,34].

We stress that comparison does not entail ignoring differences, but rather aims to reveal commonalities and contrasts within and among species to enhance our understanding of the socioecological circumstances that promote more or less equal societal structures. Such differences between human and non-human animals are driven in part by human reliance on symbolic communication (syntactic language) and a depth and complexity of cultural inheritance unequalled in other species [37,38]. Yet, many species share common mechanisms for promoting or disrupting social structures that contribute to inequality. Whereas the study of evolutionary processes in non-human animals can offer insights into factors shaping the origins of power dynamics (e.g. [39]) and cooperation (e.g. [40]) in humans, approaches used to study humans may also offer new insights and theoretical predictions that can be used in turn to study and explain patterns of equality and inequality in non-human animals [5]. In sum, we argue that cross-species comparison can provide valuable insights into the factors contributing to equality and inequality.

The empirical papers in this issue focus almost exclusively on mammalian species, including humans. We offer two primary justifications for this focus. First, the reproductive ecology of social mammals constrains the dynamics of inequality in ways that differ greatly from the possible forms it can take in many other taxonomic groups, and markedly so in social insects. Second, the mechanisms underlying unequal access to resources in humans are more comparable (whether or not they are homologous) in social mammals, including other primates. Nevertheless, we recognize that broader comparative studies might prove fruitful.

When comparing inequality in humans and other mammals, both similarities and differences are evident. Drivers of inequality that appear similar across species include resource control, kin-based politics and coalitions of both dominants and subordinates competing for power. Comparative evidence on these is found in many articles in this issue.

One factor central to inequality in human societies is the role of institutions. Some scholars define institutions quite broadly; for example, ‘locally stable, widely shared rules that regulate social interaction’ ([41], p. 326). Others adopt a narrower meaning that refers to a set of explicit roles assigned to individuals and the rules governing their behaviour (cf. [25,34]). Most would agree that the term covers both formal institutions such as legal rules and procedures, inheritance systems, political offices (and their rules of succession), marriage rules, economic regulations, and class- and caste-based systems, as well as various informal practices or norms.

Analyses of inequality in human societies, including several in this issue, often focus on whether and when inequality becomes institutionalized. All human societies, and indeed those of other social species, exhibit *achieved* differences between individuals in status, skill, and influence or power over others, including at minimum differences due to age and strength. However, these forms of inequality, even though recurrent, wax and wane with their underlying individual attributes; they are easily reversed and are not passed to others via institutions [42]. *Institutionalized inequality* is qualitatively different, involving codified differences in power and wealth that are *ascribed* to individuals via inheritance (e.g. hereditary slavery, aristocracy) or some other institutional procedure (e.g. priesthood) [9]. Most anthropologists and archaeologists believe that institutionalized inequality was absent for most of the 300 or so millennia that *Homo sapiens* has existed, as discussed in the following subsection.

Social interactions in non-human animals are often also structured by roles and patterns analogous to institutions, such as dominance hierarchies, alliances, leadership roles, territoriality and mating systems. Such structures are particularly evident in animal societies in which hierarchical positions are passed on from one generation to another via arbitrary social conventions (e.g. nepotistic inheritance) to reinforce intergenerational legacies of inequality [43]. Matrilineal inheritance structures profoundly influence resource access, survival and reproduction in non-human animals [7], but matrilineal human societies, such as that of Mosuo, possess striking similarities in how power and access is transferred among maternal lines [27]. Like humans, other mammals also possess countering mechanisms such as inequity aversion, peacekeeping, forgiveness and sharing food with non-kin [44–48] to reduce inequality [7]. Moreover, variation in dominance structures across mammals exhibits minimal phylogenetic constraints, revealing greater flexibility in this social trait than previously assumed [7]. Nevertheless, institutions clearly have much greater elaboration and variability in our species compared to any other single non-human species. Presumably, this is due to much higher rates of cultural transmission and resultant behavioural diversification, as well as cumulative cultural evolution [37], resulting from the high-volume information flow made possible by language [49]. Comparative study of these processes across the Tree of Life could uncover the conditions promoting more or less equal societies, revealing the general processes that (de)stabilize social structures that contribute to inequality.

Although human populations do certainly exhibit reproductive skew [50], extreme forms of reproductive suppression and altruism such as in mole rats [20] and social insects [51] have little human counterpart. One key difference in the human case is attributed to enhanced paternity certainty and resultant paternal investment, resulting in a major expansion of kinship ties and the option of patrilineal as well as matrilineal networks and inheritance pathways [27,28,52,53]. In addition, ecological changes in the hominin lineage may have favoured paternal provisioning [54]. Data in this special issue also highlight that patterns of reproductive skew in other species are by no means fixed or static but rather vary from population to population within species. For example, reproductive skew among our closest relatives, chimpanzees and bonobos, also varies within species and among communities [21]. These patterns reflect adaptive variability and the flexible nature of power systems in mammalian societies.

In sum, differences between humans and other mammals are apparent at the levels of mechanisms, intensity and dynamics of inequality, while similarities appear to lie in evolutionary ecology principles that account for these patterns. Analysis of these factors can offer new insights into the mechanisms promoting diversity in social structures.

### (c) The late blooming of persistent institutionalized inequality

Many small-scale human societies are relatively egalitarian, meaning that status and power differentials *within age and gender categories* are muted and primarily achieved rather than ascribed, and access to subsistence resources is equalized through sharing and other means. This contrasts with many social mammals, including some of our closest primate relatives [7,55]. For non-human species, dominance due to size or strength is best classified as achieved, whereas dominance due to mother's rank is ascribed. Some argue that human egalitarianism is due to countermeasures such as active resistance to domination or collective punishment of aggrandizing behaviour [56–58], while others point to ecological drivers such as risk-pooling and gains from cooperation [59,60].

Be that as it may, the archaeological record indicates that institutionalized inequality, as measured typically by grave goods, as well as by variations in residential size and other architectural signatures, is rare until Holocene times (beginning roughly 11 millennia ago) [61–63] although some contest this [64], and periodic episodes of inequality that came and went in the more distant past may prove to have been more common than currently documented. But it is only in the past few millennia that non-egalitarian and even markedly stratified systems have replaced nearly all such societies. The near absence of institutionalized differences in wealth and power for most of the history of our species raises the question of what changed. Several papers in this special issue offer important clues [10,14,18]. The development and spread of agriculture certainly accounts for some of the temporal dynamics of institutionalized inequality, but its absence in low-intensity ‘horticultural’ societies [65], muted presence even among some agriculturally dependent state-level societies [66–68] and multiple cases of non-egalitarian hunter–gatherers [69,70] indicate it cannot be the only, or perhaps even the main, explanation. In terms of timing, the high-amplitude high-frequency climate fluctuations of the Pleistocene, and their amelioration in the Holocene, suggests a historically contingent answer for the late emergence of inequality [9,71]. In particular, Holocene climate amelioration increases the economic defensibility of high-quality resource patches by dominants, who can transmit these holdings to descendants as well as offer access to subordinates in exchange for labour and other services [9,10].

The asymmetries in bargaining power that arise from controlling highly productive resources (especially arable land) in turn fuel economic specialization and exchange, further cementing institutionalized inequality [16,66,72]. However, particular ecological circumstances can limit economic productivity even in Holocene climates, thus allowing small-scale relatively egalitarian systems to persist into the contemporary historic period [73]. This undercuts misinterpretations of Holocene history as a uniform process and highlights the multifaceted conditions that are necessary for the emergence and persistence of inequality.

### (d) Biological consequences of inequality

There is considerable research on the effects of inequality on various biologically significant dimensions, including health and mortality, nutrition or food intake, status or social influence, and reproductive success. A recent review [74] illuminates the ways in which social factors shape health and survival in humans and other social mammals. Both theoretical and empirical work implicates income inequality (measured by the Gini index) as fostering low levels of trust and high levels of violence and mortality, even holding average income constant in humans [75–78]. In non-human animals, biologists often measure inequality in terms of hierarchy strength, which influences an individual's priority of access to resources that contribute to variation in reproductive success and survival [5–7]. These measures allow for comparisons among societal structures to help identify which ecological conditions and historical factors contribute to more or less equal societies.

The association between hierarchy and health is evident for many social mammals, from primates [79] and carnivores [80] to ground squirrels [81]. In human societies, this pattern is well documented for modern, large-scale societies [25,82]. For small-scale societies, the evidence is mixed (cf. [83,84]), although those subject to colonial and racist regimes clearly suffer from huge inequalities in health care access and outcomes [85,86].

Differences in both material and relational wealth impact social influence, although effects can clearly flow in both directions [21,32]. Effects of unequal wealth and power according to gender can be quite complex in both humans [27,28] and other species [20,87,88]. The uniquely developed degree of paternal investment and kinship reckoning in humans noted above creates its own set of variations involving matrilineal versus patrilineal inheritance of wealth and social status.

Reproductive success, closely related as it is to fitness, is of obvious significance in evolutionary analyses. While much variation in reproductive success can be due to individual circumstances, some of it certainly falls within the socially structured variation we define as inequality [89]. The varied forms and dynamics this can take are amply covered in various papers in this issue [7,11,13,20,21,31,32].

A comparative approach has the potential to reveal factors favouring or countering inequality across social mammals, as well as patterned consequences of inequality. The documentation and analysis of variation in inequality across multiple species by no means portrays inequality as invariable or inevitable. To the contrary, such research demonstrates the complexity of social dynamics, and their effects on wealth distributions in a range of ecological circumstances.

### (e) Social and political implications

Various critics have argued that sharing concepts between biology and social sciences (in either direction) ‘naturalizes’ phenomena such as inequality, hierarchy and gender roles—and in so doing makes them seem inevitable, thus reinforcing the oppressive status quo [90,91]—or otherwise conceals socially constructed aspects of inequalities [92,93]. To this we offer two responses. First, something being ‘socially constructed’ does not entail that there is no role for ecological or evolutionary factors (or does so only in extreme versions of social constructionism, a form of Cartesian dualism we reject). Second, the kinds of social or behavioural phenomena examined in this issue are not like eye colour or blood type, but phenotypically plastic traits, and in many cases conditional strategies [49] that help to adapt behaviour to current context. In such cases, the evolved feature is not the behaviour or other phenotypic expression, which can change rapidly and dramatically, but the underlying strategy or reaction norm [94–96].

More moderate critiques might hold that evolutionary analyses of human behaviour may have some scientific validity but are too easily distorted by others to justify or reinforce existing oppression. In effect, they propose that the costs (in potential societal harm) outweigh the benefits (in scientific insight and applied potential). Although we see some merit in this position, we feel it should only stand in cases where the insights have a weak basis, and the potential harm is significant and highly probable. Furthermore, ceding evolutionary analysis to those who valorize status quo inequalities is unwise; pretending there is no evolutionary or ecological basis to inequality in cases where evidence clearly supports such inference is intellectually dishonest, and can potentially strengthen regressive agendas. Indeed, if we wish to identify ways to reduce inequality—whether based on class, gender, race, or some other attribute—we must first understand the underlying causes, which evolutionary ecology is primed to contribute to. It is our sincere hope that the body of work set forth in this theme issue will help to elucidate the mechanisms contributing to wealth disparities to offer new insights for mitigating their harmful effects.

The view taken by most papers in this issue is that enduring, systematic differences in wealth and power arise out of long-term socioecological dynamics, including competition, resource transfers within and between generations, and collective action, as well as chance events. This view is closer to historical materialism (the theory of social change developed by Marx & Engels [97] and Cohen [98]) than to any form of social Darwinism or genetic determinism. Instead, our focus is on the ecological circumstances that favour or resist inequality, and how these processes can accumulate over time in human and non-human societies. This approach does not deny agency, but rather places goals and preferences—and constraints on those goals and preferences—within a complex framework that is ultimately subject to evolutionary analysis, whether biological or cultural [99]. In sum, analysing the causes and consequences of inequality (or any other phenomenon) does not entail justifying these as right or inevitable. To the contrary, deeper understanding is often necessary to mitigate or eliminate them.

## 3. Conclusion and prospects

In this issue, various research projects analyse how multifaceted environmental and social dynamics interact to allow or discourage the emergence of inequality in wealth, power and well-being. Further progress in disentangling drivers of inequality as well as its diverse effects will require both theoretical advances (e.g. [30]) and increasingly sophisticated empirical research that integrates data from multiple disciplines [100–102]. Although structural inequality is widespread in social species, the research reported in this issue demonstrates that it would be a mistake to view it as an inevitable or invariable outcome of reproductive competition or natural selection more generally—a point developed further elsewhere in this issue [7].

There is no question that research in both biology and evolutionary social science can be repurposed to support conservative or regressive views. White nationalists and neo-Nazis, for example, sometimes cite genetic research or Darwinian theory to advance their racist and xenophobic agendas [103,104]. However, this argument cuts both ways, as behavioural biology and evolution can be used to support progressive arguments [105–107]. Additionally, regressive political views can find comfort in claiming human exemption from biological evolution [108,109]. Be that as it may, we agree with those who hold that progressive politics can be quite compatible with efforts to use evolutionary and ecological concepts to understand human behavioural variation [110,111]. Evolutionary social scientists frequently contribute substantive critiques of racism, sexism, ethnocentrism, and other oppressive ideologies and practices [87,112,113], and empirical evidence refutes the claim that they are more likely to hold regressive views [114,115].

We acknowledge the ways in which unconscious bias and positionality can affect our research, and the potential for others to wrongfully co-opt such research for their own purposes. However, we argue it would be a mistake to abandon such research out of these concerns. Indeed, failing to understand the underlying drivers of inequality, as well as mechanisms that counter it, might well trap us in a position where we can do little to reduce it. To that end, contributions in this special issue highlight factors that influence (in)equality across mammalian societies, advancing our understanding of its causes and consequences that are common as well as unique. Our hope is that this helps advance a unifying evolutionary ecological framework regarding (in)equality.

## Data Availability

This article has no additional data.
